# Effects of praziquantel on common carp embryos and larvae

**DOI:** 10.1038/s41598-022-21679-2

**Published:** 2022-10-14

**Authors:** Josef Velisek, Eliska Zuskova, Jan Kubec, Marie Sandova, Alzbeta Stara

**Affiliations:** South Bohemian Research Center of Aquaculture and Biodiversity of Hydrocenoses, Research Institute of Fish Culture and Hydrobiology, University of South Bohemia in Ceske Budejovice, Faculty of Fisheries and Protection of Waters, Zatisi 728/II, 389 25 Vodnany, Czech Republic

**Keywords:** Biochemistry, Environmental sciences, Health care

## Abstract

This study aimed to assess the toxicity of praziquantel (anthelmintic drug) in different developmental stages of common carp (*Cyprinus carpio*) based on mortality, early ontogeny, growth, oxidative stress, antioxidant enzymes, histology and behaviour. Praziquantel at all tested concentrations ranging from 1 to 4 mg/L showed no significant adverse effects on mortality, the early ontogeny and behaviour locomotory (activity, moved distance and velocity) of carp after 35-day exposure. Concentrations of 3 and 4 mg/L caused significantly (*P* < 0.01) lower growth, total superoxide dismutase and catalase activities compared with controls. Praziquantel is safe for the early life of carp in concentrations ≤ 2 mg/L.

## Introduction

Praziquantel [2-(cyclohexylcarbonyl)-1,2,3,6,7,11b-hexahydro-4H-pyrazino (2,1-a) isoquinolin-4-one] is a pyrazinoisoquinoline medication developed for the treatment of helminth infections in humans and domestic animals^1,2^. Praziquantel is considered as useable antiparasitic compound against fish platyhelminths^2–6^). For praziquantel, the maximum residual limit in fish in the EU has not been determined, unlike in some non-EU states and can be used only in ornamental and non-food fish. Praziquantel lacks registration in aquaculture practices, though a potential selection of such compounds is possible in aquaculture under the ‘off-label’ cascade (Council Directive 90/676/EEC, Directive 2001/82/EC and Commission Regulation 37/2010). For those cases, a standard withdrawal time of 500-degree days is enforced to secure consumer safety. Nevertheless, praziquantel could be an appropriate candidate for parasite elimination from fish before transfer to the final rearing location or from infected non-food broodfish^7,8^. As reported by many studies, praziquantel can be used in the form of a bath or oral administration to control target parasites of fish^4,6,7,9–12^. Recommended concentrations of therapeutic baths range from 0.25 to 50 mg/L depending on the bath duration and species of the treated parasites. Usually, a bath treatment typically involves a low concentration (up to 10 mg/L) of praziquantel for an extended time. In contrast, a dip utilises a high concentration (tens of mg/L) of praziquantel for a shorter time. Contrary, oral praziquantel administration ranges from 50 to 200 mg/L for single doses and from 7 to 75 mg/L for repeated multiple doses^7^.

The 24hLC50 dose of praziquantel in the fry of the North African Catfish (*Clarias gariepinus*) is 13.4 mg/L^13^, in golden shiner (*Notemigonus crysoleucas*) is 55.1 mg/L, and in grass carp (*Ctenopharyngodon idella*) is 49.7 mg/L^14^. Studies of the praziquantel efficiency to fish indicate that it can cause mortality^14^, affect haematological and biochemical profile^15^ and enhance some specific and non-specific immune parameters^16^. However, scientific sources lack data about the particular effects of praziquantel on the early life stages of carp.

Common carp (*Cyprinus carpio*) is a major farmed species in Asia and European freshwater aquaculture and contributes around ~ 4.67 million metric tons on a global scale the Czech Republic, Poland, Hungary, and Germany fisheries are producing 80% of the carp in the European Union^17^. Newly hatched carp larvae constitute a particularly critical and sensitive life stage, since at hatching the embryos lose their protective membrane and are fully exposed to potential toxicants. Carp was selected because it is the most frequently bred fish in the Czech Republic. Newly hatched carp larvae constitute a particularly critical and sensitive life stage, since at hatching, the embryos lose their protective membrane and are fully exposed to potential toxicants^18^. Common carp was selected because it is the most frequently bred fish in the Czech Republic. Little information is available on the toxicity of praziquantel to fish, and the safety margin between a treatment rate and toxic doses is unknown for most fish species. The study aimed to assess the effects of the antiparasitic drug praziquantel on early-life stages of common carp (*Cyprinus carpio*), namely the impact on (1) mortality; (2) growth rate; (3) ontogenetic development; (4) behaviour; (5) oxidative stress response and antioxidants biomarkers; and (6) histological structure.

## Materials and methods

### Chemicals and chemical analysis

Praziquantel was obtained from Ecological Laboratories Inc., USA. Ethanol 96% was purchased from Merck KGaA, Germany. Praziquantel concentrations were checked daily before and after the bath renewal by ultrahigh-performance liquid chromatography (UHPLC) using the method of Zrncic et al.^19^.

### Experimental animals

Fertilised carp eggs were obtained from a hatchery of the University of South Bohemia in Ceske Budejovice, Faculty of Fisheries and Protection of Waters, Czech Republic. Eggs were fertilised by the methods described by Kocour et al.^20^.

All the methods used in the present study followed relevant guidelines and regulations. Also, the competent authority (Ethical Committee for the Protection of Animals in Research of the University of South Bohemia, FFPW Vodnany) approved the fish sampling and protocols of the present study and reporting herein follows the recommendations in the ARRIVE guidelines^21^.

### Experimental protocol

The investigation was carried out using the modified No. 210 OECD test^22^. At 24 h post fertilisation, 100 fertilised eggs were placed into each of eighteen glass basins with the praziquantel solution. The concentrations of praziquantel used were 1.0 mg/L (P1 group), 2.0 mg/L (P2 group, concentrations used for antiparasitic bath, Noga^23^), 3.0 mg/L (P3 group) and 4.0 mg/L (P4 group).

Two other groups were used as contrast groups, a control group (C) exposed to clean, fresh water and an ethanol group (CE) used as solvent control (contained 0.8 ml/L ethanol, this concentration was used for the highest concentration of praziquantel). The stock solution of praziquantel was prepared by adjusted the required concentration of praziquantel dissolved in ethanol (5 mg/mL). Ethanol was used as a solvent due to the low solubility of praziquantelin water. Each experimental condition was examined in triplicate.

The solution for each treatment was renewed daily. Daily mortality, morphological anomalies, behaviour, oxygen saturation, pH and dead carp were monitored. Water quality parameters were as follow: temperature 21.3 ± 0.7 °C, dissolved oxygen > 4.56 mg/L, pH 7.76–8.05, acid neutralization capacity (ANC_4.5_) 0.68 mmol/L; chemical oxygen demand (CODMn) 0.84 mg/L, total ammonia 0.010 mg/L; suma Ca^2+^ + Mg^2+^ 5.87 mg/L. The temperature was measured hourly using Minikin loggers (Environmental Measuring Systems, Brno, Czech Republic).

From day 6, carp larvae were fed ad libitum with *Artemia salina* nauplii twice a day. On days 7, 14, 21, 28, and 35, 6 carps in each experimental group were collected to examine ontogenetic development and growth. The toxicity test was terminated after 35 days when tested animals were analysed for behavioural patterns. After behaviour analysis, animals were euthanised (MS222, 250 mg/L), weighed and stored in tubes at − 80 °C until further biochemical analyses.

### Early ontogeny

Developmental periods were defined according to Penaz et al.^24^, who described nine embryonic (E1–E9), six larval (L1–L6), and two juvenile stages (J1–J2) of common carp.

### Growth rate

The total length (TL) was individually measured by stereomicroscope using a filar micrometre. After removing excess water on a filter paper, the whole body weight was determined using a Mettler-Toledo (Greifensee, Switzerland) analytical balance to the nearest 0.1 mg. The mean specific growth rates (SGR) of experimental groups were calculated for the period from day 7 (the first sampling) to day 35 (end of the exposure). Exposed groups were compared with control using the method described by OECD^25^. The following formula of SGR was used:

$$ SGR = \frac{{\overline{{\ln w_{2} }} - \overline{{\ln w_{1} }} }}{{t_{2} - t_{1} }} \cdot 100 $$

where SGR is the mean specific growth rate in the group, w_1_ is the mass of one fish at time t_1_ individually (µg), w_2_ is the mass of one fish at time t_2_ individually (µg), $\overline{{\ln w_{1} }}$ is the mean value of the $\ln w_{1}$ values, $\overline{{\ln w_{2} }}$ is the mean value of the $\ln w_{2}$ values, t_1_ is the time (day 7)—first sampling time, t_2_ is the time (day 35)—end of exposure.

The inhibition of specific growth rate (I) in each experimental group was calculated as follows.

$$ I_{x} \left[ \% \right] = \frac{{SGR_{x} (control) - SGR_{x} (group)}}{{SGR_{x} (control)}} \cdot 100 $$

where $I_{x}$ is the inhibition of specific growth in the selected experimental group after $x$ days of exposure, $SGR_{x} (control)$ is the mean specific growth rate in the control group, $SGR_{x} (group)$ is the mean specific growth rate in the chosen experimental group.

Fulton’s weight condition factor (FWC) was calculated for each experimental group at every sampling time:

$$ FWC = \frac{{W \cdot 10^{5} }}{{TL^{3} }} $$

where FWC is the Fulton’s weight condition factor, *W* is the mass in selected experimental group (g), and TL is the total length in selected experimental group (mm).

### Locomotory behaviour

At the end of the experiment, a dozen of juvenile carp for each experimental group were placed individually in 6-well plates with 15 ml of aged tap water with a constant water temperature of 20.2 °C for 5 min of acclimatisation and subsequently video-monitored for 15 min. The DanioVision (Noldus, Wageningen, Netherlands) performed a locomotion assay, which allows the motility of carp larvae to be evaluated through a system equipped with an observation chamber that monitors the locomotory behaviour of multiple individuals at the same time under controlled conditions. The data were obtained using the behavioural software EthoVision^®^ XT 13 (Noldus, Wageningen, Netherlands) directly processed the live-tracked videos of the fish movement. The measured variables were activity (%), distance moved (cm) and mean velocity (cm/s).

### Oxidative stress and antioxidants biomarkers

Biomarkers were evaluated in the surviving carp of experimental groups after 35 days of exposure. Whole-body samples were immediately frozen and stored at − 80 °C for analysis. Frozen samples were weighed and homogenised with an Ultra Turrax homogeniser (Ika, Germany) using 50 mM potassium phosphate buffer (1:10, w/v), pH 7.0, containing 0.5 mM EDTA according to methods Stara et al.^26^. Obtained supernatant was used for further analysis.

Lipid peroxidation as TBARS was estimated spectrophotometrically according to Lushchak et al.^27^. Total superoxide dismutase (SOD) activity was estimated spectrophotometrically using the method of Marklund and Marklund^28^. The catalase (CAT) activity was measured by the spectrophotometric following the method of Beers and Sizer^29^. The glutathione S-transferase (GST) activity was measured using the method of Habig et al.^30^. Glutathione reductase (GR) activity was determined spectrophotometrically, measuring NADPH oxidation at 340 nm^31^. Reduced glutathione (GSH) level was assayed using the method of Tipple and Rogers^32^. Protein levels were estimated spectrophotometrically by the method of Bradford^33^ using bovine serum albumin as a standard.

### Histology

Histological examination was conducted in experimental groups after 35 days of exposure. Six juvenile carps from each experimental group and controls were placed in 10% formalin. Samples were prepared with standard histological techniques^34^, embedded in paraffin, stained with haematoxylin and eosin, and examined by light microscopy.

### Statistical analysis

Differences in cumulative mortality between experimental groups and controls were assessed using Chi square test. Before analysis, all measured variables were checked for normality (Kolmogorov–Smirnov test) and homoscedasticity of variance (Bartl’tt's test). Then, a one-way ANOVA was employed to determine whether there were significant differences in measured variables among experimental groups if those conditions were satisfied. When a difference was detected (*P* < 0.05), the Tukey Unequal N HSD test was applied. If the conditions for ANOVA were not satisfied, a non-parametric test (Kruskal–Wallis) was used.

## Results

### Mortality, hatching

Hatching began 3 days after the onset of exposure. The eggs in all groups hatched by day 6. No significant adverse effects of praziquantel on hatching were observed. No significant differences were found in cumulative mortality among groups. Accumulated mortality in the group exposed to praziquantel in concentrations of 1 mg/L (P1), 2 mg/L (P2), 3 mg/L (P3) and 4 mg/L (P4) was 9, 10, 11 and 10%, and in ethanol control (CE), control (C) was 11 and 9% respectively.

### Early ontogeny

**Figure 1:**
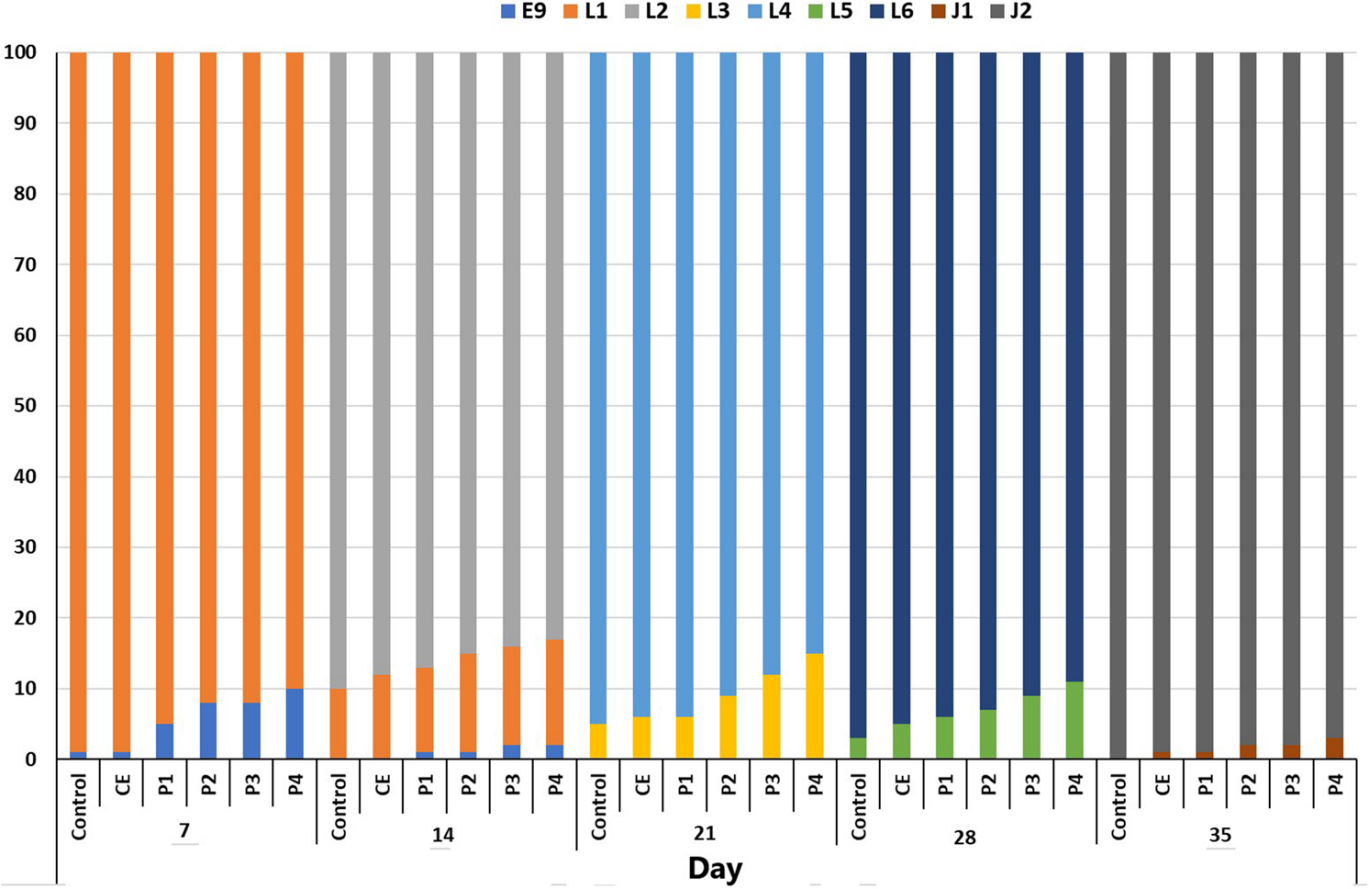
Developing stages of common carp (*Cyprinus carpio*) during 35 days of exposure to praziquantel. *E* embryonic stage, *L* larval stage, *J* juvenile stage.

The developmental stages observed at the sampling times in all tested groups and controls are presented in Fig. 1. Praziquantel at all tested concentrations, showed no significant adverse effects on the early ontogeny of carp. At the end of the experiment, the carp in control (100%), CE (99%), P1 (99%), P2 (97%), P3 (95%) and P4 (94%) reached the juvenile stage (J2).

### Growth

**Figure 2:**
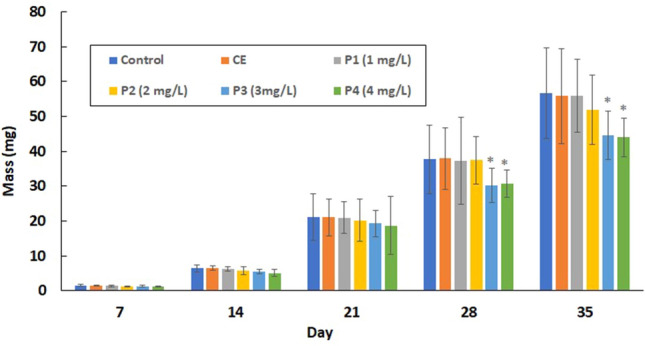
Mean weight of common carp (*Cyprinus carpio*) embryo, larvae and juveniles after praziquantel exposure. SD = standard deviation. *Significantly (*P* < 0.01) difference between experimental and control groups. Groups: CE (solvent control, contained 0.8 ml/L ethanol), P1 (praziquantel 1.0 mg/L), P2 (praziquantel 2.0 mg/L), P3 (praziquantel 3.0 mg/L) and P4 (praziquantel 4.0 mg/L).

**Figure 3:**
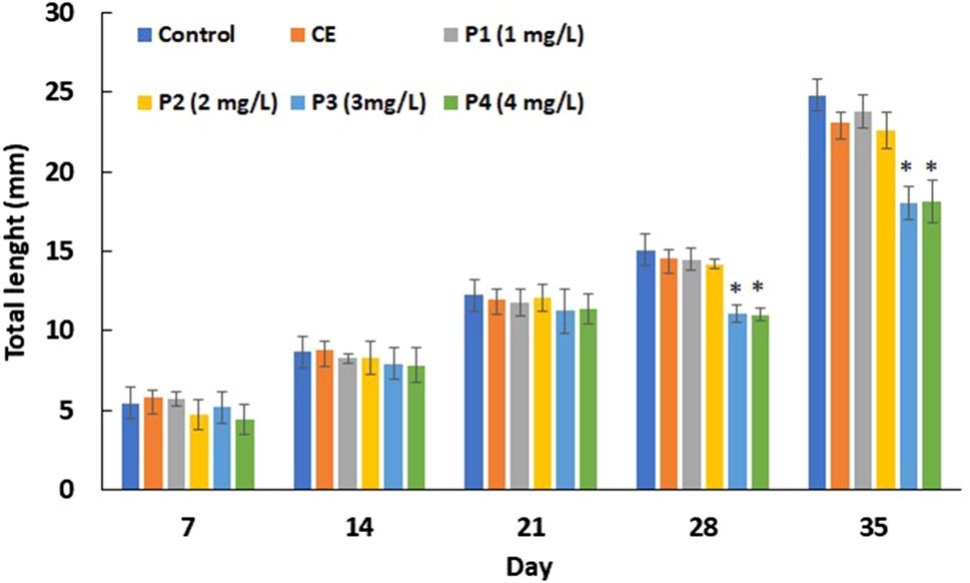
The total length of common carp (*Cyprinus carpio*) embryo, larvae and juveniles after praziquantel exposure. SD = standard deviation. *Significantly (*P* < 0.01) difference between experimental and control groups. Groups: CE (solvent control, contained 0.8 ml/L ethanol), P1 (praziquantel 1.0 mg/L), P2 (praziquantel 2.0 mg/L), P3 (praziquantel 3.0 mg/L) and P4 (praziquantel 4.0 mg/L).

Beginning on day 28 of exposure, carp exposed to the two highest praziquantel concentrations (3 and 4 mg/L) showed significantly (*P* < 0.01) lower mass (Fig. 2) and total length (Fig. 3) compared with controls. The FWC values of carp are given in Table 1. From 28th day of exposure, the FCF values were significantly (*P* < 0.01) lower in the two highest praziquantel concentrations (3 and 4 mg/L) compared with controls.

**Table 1:** Mean Fulton’s condition factor for common carp during exposure to praziquantel.

Times (day)	Control	CE	Praziquantel			
			P1 (1 mg/L)	P2 (2 mg/L)	P3 (3 mg/L)	P4 (4 mg/L)
	Mean ± SD	Mean ± SD	Mean ± SD	Mean ± SD	Mean ± SD	Mean ± SD
7	1.02 ± 0.38	0.92 ± 0.32	0.96 ± 0.13	0.95 ± 0.26	0.96 ± 0.26	1.00 ± 0.28
14	0.99 ± 0.20	0.99 ± 0.17	1.16 ± 0.30	1.06 ± 0.33	1.22 ± 0.51	1.15 ± 0.43
21	1.13 ± 0.13	1.16 ± 0.42	1.15 ± 0.22	1.07 ± 0.23	1.11 ± 0.15	1.02 ± 0.22
28	1.22 ± 0.12	1.21 ± 0.10	1.18 ± 0.20	1.15 ± 0.20	1.02 ± 0.52*	0.95 ± 0.10*
35	1.34 ± 0.18	1.19 ± 0.10	1.21 ± 0.17	1.09 ± 0.16	0.94 ± 0.09*	0.82 ± 0.16*

*Experimental groups significantly (*p* < 0.01) different from the control group.

SD = standard deviation. Groups: CE (solvent control, contained 0.8 ml/L ethanol), P1 (praziquantel 1.0 mg/L), P2 (praziquantel 2.0 mg/L), P3 (praziquantel 3.0 mg/L) and P4 (praziquantel 4.0 mg/L).

Specific growth rates and inhibition of growth of carp exposed to praziquantel are given in Table 2. Compared to control, inhibition of carp growth was 2.5, 4.4, 12.4 and 16.9% in groups P1, P2, P3 and P4, respectively.

**Table 2:** Growth indices of carp during 35 days exposure of praziquantel.

Fish group	Control	CE	Praziquantel			
			P1 (1 mg/L)	P2 (2 mg/L)	P3 (3 mg/L)	P4 (4 mg/L)
m_7_	1.53 ± 0.21	1.47 ± 0.21	1.40 ± 0.23	1.35 ± 0.10	1.35 ± 0.24	1.23 ± 0.14
m_35_	56.65 ± 13.06	55.03 ± 13.53	55.95 ± 10.44	48.40 ± 9.01	46.62 ± 9.89*	45.02 ± 10.58*
SGR	13.29	12.86	12.96	12.71	11.64	11.04
I (%)	–	3.24	2.48	4.36	12.42	16.93

m_7_, m_31_ = Mean carp weight in group after 7 and 35 days exposure (Mean ± SD, mg); SGR = specific growth rate in group after 28 days exposure; I = inhibition of specific growth in selected group after 28 days exposure; SD = standard deviation. *Significantly (*P* < 0.01) difference between experimental and the control group. Groups: CE (solvent control, contained ethanol 0.8 ml/L), P1 (praziquantel 1.0 mg/L), P2 (praziquantel 2.0 mg/L), P3 (praziquantel 3.0 mg/L) and P4 (praziquantel 4.0 mg/L).

### Locomotory behaviour

The control individuals did not exhibit any significant alternations in the moved distance (H = 4.045, *P* > 0.05) compared to P1, P2, P3, and P4-exposed juveniles. Similarly, no significant differences in the moved distance in groups P3 and P4 compared to the ethanol group (H = 0.655, P > 0.05). No significant differences were detected in the mean of larvae velocity (H = 8.175, *P* > 0.05), and distance moved (H = 6.102, *P* > 0.05) between both control groups and Praziquantel-exposed animals. Data are shown in Table 3.

**Table 3:** Moved distance, velocity, and cumulative movement of juvenile carp exposed to praziquantel concentration and in control fish. Groups: CE (solvent control, contained 0.8 ml/L ethanol), P1 (praziquantel 1.0 mg/L), P2 (praziquantel 2.0 mg/L), P3 (praziquantel 3.0 mg/L) and P4 (praziquantel 4.0 mg/L). Data are shown as mean ± standard deviation.

Fish group	Praziquantel					
	Control	CE	P1 (1 mg/L)	P2 (2 mg/L)	P3 (3 mg/L)	P4 (4 mg/L)
Moved distance (cm)	435.6 ± 128.4	569.9 ± 109.7	454.9 ± 60.1	486.1 ± 180.1	532.5 ± 92.7	519.4 ± 192.1
Velocity (cm/s)	0.48 ± 0.14	0.63 ± 0.12	0.51 ± 0.07	0.54 ± 0.20	0.59 ± 0.10	0.58 ± 0.21
Cumulative movement (%)	48.3 ± 10.4	54.1 ± 10.7	48.3 ± 7.3	50.8 ± 14.5	58.4 ± 10.5	52.5 ± 14.2

### Oxidative stress and antioxidant response

The effects of praziquantel exposure on the oxidative stress and antioxidant response of juvenile carp are given in Table 4. Carp exposed to the two highest praziquantel concentrations (3 and 4 mg/L) showed significantly (*P* < 0.01) lower total SOD and CAT activity compared to the controls. No significant differences among groups were seen in TBARS, GST, GR or GSH activity.

**Table 4:** jscsccb.

Group	Control	CE	Praziquantel			
			P1 (1 mg/L)	P2 (2 mg/L)	P3 (3 mg/L)	P4 (4 mg/L)
TBARS	0.432 ± 0.021	0.421 ± 0.018	0.398 ± 0.024	0.441 ± 0.034	0.456 ± 0.036	0.478 ± 0.044
SOD	0.264 ± 0.029	0.244 ± 0.031	0.221 ± 0.026	0.219 ± 0.016	0.165 ± 0.017*	0.155 ± 0.013*
CAT	0.564 ± 0.103	0.601 ± 0.145	0.531 ± 0.102	0.503 ± 0.131	0.401 ± 0.087*	0.356 ± 0.092*
GST	1.335 ± 0.245	1.189 ± 0.209	1.298 ± 0.263	1.305 ± 0.299	1.164 ± 0.310	1.109 ± 0.384
GR	0.356 ± 0.112	0.389 ± 0.123	0.341 ± 0.131	0.386 ± 0.105	0.401 ± 0.129	0.409 ± 0.133
GSH	10.11 ± 1.25	11.23 ± 1.58	10.89 ± 1.12	11.05 ± 2.05	11.36 ± 2.45	11.41 ± 2.08

The effect of praziquantel on oxidative stress biomarker (thiobarbituric acid reactive substances—TBARS, nmol/mg protein) and antioxidant enzymes (superoxide dismutase—SOD, nmol NBT/min/mg protein; catalase—CAT, µmol H_2_O_2_/min/mg protein; glutathione S-transferase—GST, nmol/min/mg protein; glutathione reductase—GR, nmol NADPH/min/mg protein; reduced glutathione—GSH, nmol GSH/mg protein) in the homogenate of carp. *Significantly (*P* < 0.01) difference of experimental groups compared to the control.

### Morphological abnormalities and histology

Morphological abnormalities consisting of curvature of the spine (spine shortening) and yolk sac oedema were observed in less than 2% of the fish in all praziquantel exposed groups as well as the control group. These anomalies can be considered spontaneous occurrences.

**Figure 4:**
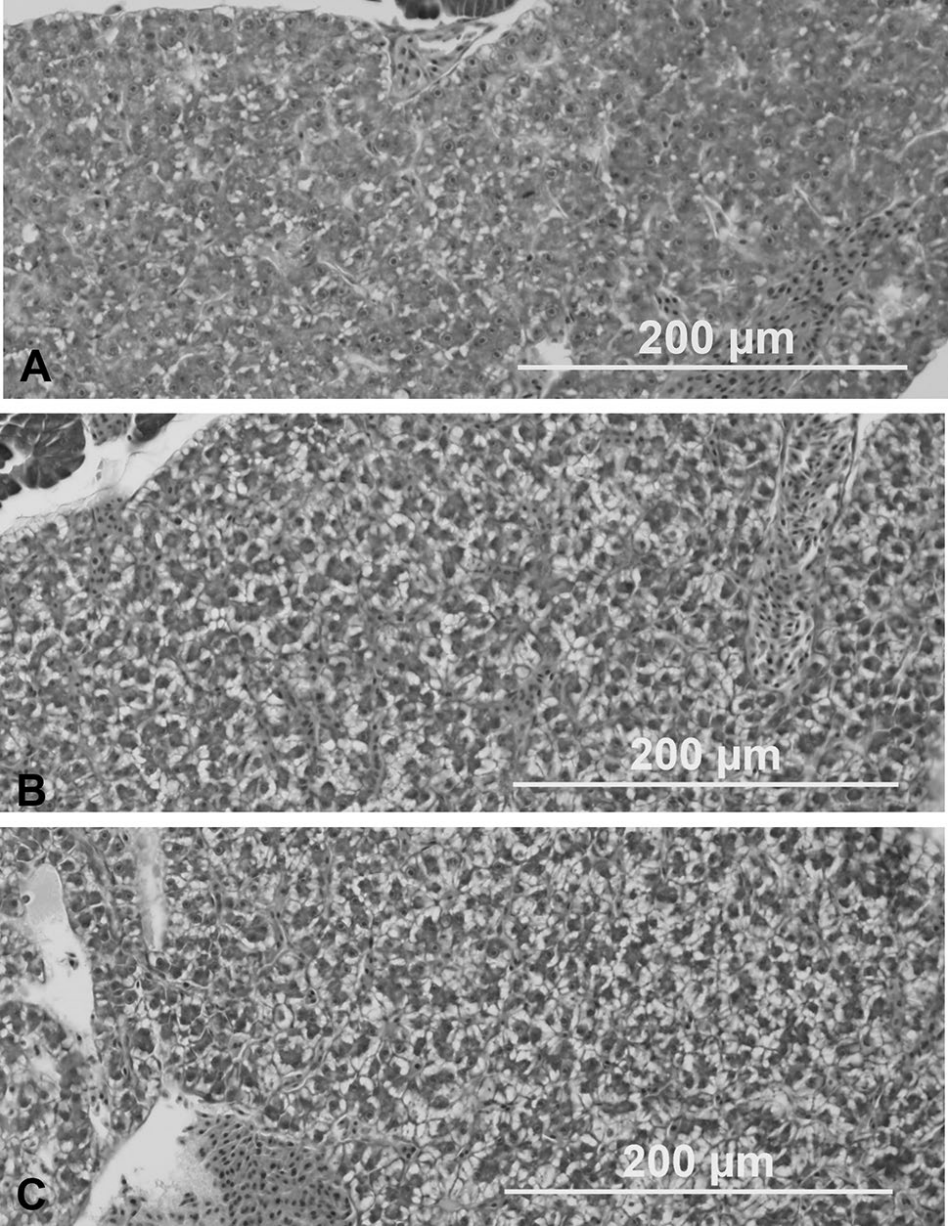
Liver of juvenile carp (*Cyprinus carpio* L.) after 35 days praziquantel exposure. Haematoxylin and eosin, × 200. (**A**) Control group; (**B**) control ethanol group (ethanol 0.8 ml/L); (**C**) Experimental group P1 (praziquantel 1.0 mg/L dissolved in ethanol). See the extensive steatosis (abnormal lipids retention) consisting of steatotic droplets of varying sizes in pictures (**B**) and (**C**).

Histological examination of the liver revealed extensive steatosis associated with the loss of cellular shape and the presence of lipid inclusions in hepatic cells. Described pathologies were found in fish within the experimental groups (P1–P4), and in the control ethanol group (CE). The liver from the control group (C) manifests only a low degree of dystrophy (Fig. 4).

No histopathological changes were demonstrated in the heart, gills, skin, cranial and caudal kidney and intestine following exposure to praziquantel.

## Discussion

Some Monogeneans, trematodes and cestodes can cause serious problems in intensive aquaculture, necessitating early diagnosis, as well as a rapid and effective treatment^23,35^. Praziquantel is a useful chemotherapeutic against helminths of captive fish. Early life stages of fish are often the most sensitive to toxic effects of xenobiotics than older stages of fish. While we have information on the toxicity of praziquantel to juvenile and adult fish, little is known about the effect of praziquantel on the early life stages of carp. The praziquantel for fish is toxic. The 24hLC50 dose of praziquantel in the fry of the North African catfish is 13.4 mg/L^13^, in grass carp is 49.7 mg/L^14^, and 96hLC50 for African catfish (*Clarias gariepinus*) is 53.52 mg/L^36^ and for barbel (*Barbus barbus*) is 28.6 mg/L^12^. In our study, was no mortality, this can be explained used by testing lower concentrations (mg/L—used for treatment bath) than are lethal (tens of mg/L) to fish.

Increasing fish production depends on feeding, handling and the absence of stress, among other factors^23,37^. Fish growth depends on several factors: species, age, genetic potential, water temperature, health, and quantity and quality of food^38^. Generally, stress conditions such as polluted aquatic environments and diseases result in decreased fish growth^23,39^. Using chemotherapeutics to control and treat parasites is one of the most critical issues in all phases of intensive fish aquaculture production^23,37,40^. In our study, praziquantel in concentrations (3 and 4 mg/L) caused lower growth than controls and inhibition of carp growth was 12.4 and 16.9% in these groups. The suppression of carp growth may be due to adverse effects of praziquantel on the metabolism, such as reducing food and nutrient uptake. Similar results were observed in the other studies with fish chemotherapeutics like formalin^37^ and copper sulphate^41^.

Newly hatched larvae constitute a particularly critical and sensitive life stage since, at hatching, the embryos lose their protective membrane and are fully exposed to a potential toxicant^38,42^. Early ontogenetic development is a sensitive biomarker for evaluating the impacts of xenobiotics or fish veterinary drugs on aquatic organisms^43–45^. In our study, praziquantel at all tested concentrations showed no significant negative effect on hatching and the early ontogeny of carp. Development inhibition might delay reproduction and increase the susceptibility of early life stages to predation. In the scientific literature, changes in early ontogenetic development are described mainly after pesticides^44–46^ and pharmaceutical compounds^46,47^ exposure.

The effects of chronic exposure to pharmacological compounds can also be expressed by analysis of locomotory or foraging behaviour^48^. We found no activity, moved distance, or velocity differences among our control and Praziquantel-exposed groups at either exposure time. On the other hand, studies by Obiekezie and Okafor^13^, Mitchell and Hobbs^14^ and Zuskova et al.^12^ found changes in the fish behaviour after praziquantel exposure, but these changes were in higher concentrations (tens mg/L). Our results indicated that the tested concentration of praziquantel would not significantly influence of juvenile carp behaviour for used concentrations. On the other hand, our study focused on possible behavioural alternations while using healthy carp juveniles, not individuals with parasitic infections. Therefore the question remains whether praziquantel-treated fish with parasite infection can be has a behavioural disruption.

Numerous chemicals, including some drugs, can evoke oxidative stress. Oxidative stress has been defined as an imbalance of oxidants and antioxidants favouring the oxidants, potentially leading to cell damage^26^. In our test, carp exposed to the two highest praziquantel concentrations (3 and 4 mg/L) showed significantly lower SOD and CAT activity than controls. Enzymes SOD and CAT systems provide the first line of defence against ROS^36^. Superoxide dismutase catalyses the dismutation of the superoxide anion radical to water and hydrogen peroxide, which CAT detoxifies. In our study, induction of SOD and CAT in the carp whole-body homogenate after exposure to praziquantel could be an adaptive response to the stress, neutralising the impact of ROS generated. Similar results were observed in the other studies with fish chemotherapeutics like formalin^49^, ivermectin^50^, copper sulphate^41^ and trichlorfon^51,52^.

In our test, extensive liver steatosis was found in all experimental groups and the ethanol control group. The lipid accumulation in hepatocytes (liver steatosis) can be associated with exposure to toxicants^53^ and increases liver vulnerability to secondary insults from cytokines or oxidative stress^54^. It is the key step in the progressive development of liver inflammation^55^. However, the described toxic effect must be attributed to solvent ethanol rather than praziquantel since similar changes in experimental groups were found in the ethanol control group. No other histopathological changes were marked following exposure to praziquantel.

## Conclusions

Many therapeutic techniques and treatments developed and used in mammals require validation before being considered safe and effective in aquatic species. In conclusion, the study demonstrated that exposure to praziquantel induced significant lower growth and changes in the antioxidant enzymes (SOD, CAT) of the early life stages of carp. These changes were found in the two highest tested concentrations (3 mg/L and 4 mg/L). Praziquantel is safe for the early life of carp in concentrations ≤ 2 mg/L. Further research is needed to understand the toxic effects observed in some species, and caution should be used when considering the use of praziquantel in a new host species.

## Data Availability

The data that support the findings of this study are available from the corresponding author upon reasonable request.

## References

[1] Shirakashi S, Andrews M, Kishimoto Y, Ishimaru K, Okada T, Sawada Y, Ogawa K (2012). Oral treatment of praziquantel as an effective control measure against blood fluke infection in Pacific bluefin tuna (*Thunnus orientalis*). Aquaculture.

[2] Kogiannou D, Nikoloudaki C, Rigos G (2021). Absorption and depletion of dietary administered praziquantel in greater amberjack Seriola dumerili. Aquaculture.

[3] Morales-Serna FN, Chapa-López M, Martínez-Brown JM, Ibarra-Castro L, Medina-Guerrero RM, Fajer-Ávila EJ (2018). Efficacy of praziquantel and a combination anthelmintic (Adecto®) in bath treatments against Tagia ecuadori and *Neobenedenia melleni* (Monogenea), parasites of bullseye puffer fish. Aquaculture.

[4] Fu G, Dong Y, Zhang X, Hu K (2020). Metabolomic profiles and pathways of praziquantel in crucian carp. Environ. Toxicol. Pharmacol..

[5] Maciel PO, Affonso EG (2021). Praziquantel against monogeneans of tambaqui (*Colossoma macropomum*). Aquacult. Int..

[6] Rigos G, Kogiannou D, Vasilaki A, Kotsiri M (2021). Evaluation of praziquantel efficacy against Zeuxapta seriolae infections in Greater Amberjack, *Seriola dumerili*. Appl. Sci..

[7] Bader C, Starling DE, Jones DE, Brewer MT (2019). Use of praziquantel to control platyhelminth parasites of fish. J. Vet. Pharmacol. Therap..

[8] Baralla E, Varoni MV, Nieddu M, Demontis MP, Merella P, Burreddu C, Garippa G, Boatto G (2020). Determination of praziquantel in *Sparus aurata* L. after administration of medicated animal feed. Animals.

[9] Farias CFS, Brandao FR, de Alexandre Sebastiao F, D Souza CM, Monteiro PC, Majolo C, Chagas EC (2021). Albendazole and praziquantel for the control of Neoechinorhynchus buttnerae in tambaqui (*Colossoma macropomum*). Aquacult. Int..

[10] Kline J, Archdeacon TP, Bonar SA (2009). Effects of praziquantel on eggs of the Asian Tapeworm *Bothriocephalus acheilognathi* S. N. Am. J. Aquacult..

[11] Sudova E, Piackova V, Velisek J, Pijacek M, Svobodova Z (2010). Efficacy testing of orally administered praziquantel to common carp naturally infected by *Caryophyllidean tapeworms* (Platyhelminthes: Eucestoda). Acta Vet. Brno.

[12] Zuskova E, Piackova V, Machova J, Chupani L, Steinbach Ch, Stara A, Velisek J (2018). Efficacy and toxicity of praziquantel in helminth-infected barbel (*Barbus barbus* L.). J. Fish Dis..

[13] Obiekezie A, Okafor N (1995). Toxicity of four commonly used chemotherapeutic compounds to fry of the African catfish, *Clarias gariepinus* (Burchell). Aquacult. Res..

[14] Mitchell AJ, Hobbs MS (2007). The acute toxicity of praziquantel to grass carp and golden shiners. N. Am. J. Aquacult..

[15] Sudova E, Piackova V, Kroupova H, Pijacek M, Svobodova Z (2009). The effect of praziquantel applied per os on selected haematological and biochemical indices in common carp (*Cyprinus carpio* L.). Fish Physiol. Biochem..

[16] Soltanian S, Vazirzadeh A, Akbary P (2018). Effect of praziquantel on hemato-immunological indices in common carp (*Cyprinus carpio*). Iran J. Sci. Technol. Trans Sci..

[17] FAO (Food and Agriculture Organization of the United Nations) (2020). The State of World Fisheries and Aquaculture. Sustainability in Action.

[18] Giulio D, Hinton DE (2008). The Toxicology of Fishes.

[19] Zrncic M, Gros M, Babic S, Kastelan-Macan M, Barcelo D, Petrovic M (2014). Analysis of anthelmintics in surface water by ultra high performance liquid chromatography coupled to quadrupole linear ion trap tandem mass spectrometry. Chemosphere.

[20] Kocour M, Gela D, Rodina M, Linhart O (2005). Testing of performance in common carp *Cyprinus carpio* L. under pond husbandry conditions. I: Top-crossing with Northern mirror carp. Aquacul. Res..

[21] ARRIVE. ARRIVE guidelines. https://arriveguidelines.org/. (2022).

[22] OECD, (Organization for Economic Cooperation and Development). Guidelines for the testing of chemicals. Section 2: Effects on Biotic Systems TG- No. 210: Fish, Early-Life Stage Toxicity Test. Paris, France, pp. 24, (2013).

[23] Noga EJ (2010). Fish Disease: Diagnosis and Treatment.

[24] Penaz M, Prokes M, Kouril J, Hamackova J (1983). Early development of the carp, *Cyprinus carpio*. Acta Sci. Nat. Brno.

[25] OECD (Organization for Economic Cooperation and Development). Guideline for Testing of Chemicals 215. Fish juvenile growth test, Paris, (2000).

[26] Stara A, Machova J, Velisek J (2012). Effect of chronic exposure to prometryne on oxidative stress and antioxidant response on early life stages of common carp (Cyprinus carpio L.). Neuroendocrinol. Lett..

[27] Lushchak VI, Bagnyukova TV, Husak VV, Luzhna LI, Lushchak OV, Storey KB (2005). Hyperoxia results in transient oxidative stress and an adaptive response by antioxidant enzymes in goldfish tissues. Int. J. Biochem. Cell. Biol..

[28] Marklund S, Marklund G (1974). Involvement of superoxide anion radical in autoxidation of pyrogallol and a convenient assay for superoxide dismutase. Eur. J. Biochem..

[29] Beers RF, Sizer IW (1952). A spectrophotometric method for measuring the breakdown of hydrogen peroxide by catalase. J. Biol. Chem..

[30] Habig WH, Pabst MJ, Jakoby WB (1974). Glutathione S-transferases. First enzymatic step in mercapturic acid formation. J. Biol. Chem..

[31] Carlberg I, Mannervik B (1975). Purification and characterisation of flavoenzyme glutathione reductase from rat liver. J. Biol. Chem..

[32] Tipple TE, Rogers LK (2012). Methods for the determination of plasma or tissue glutathione levels. Meth. Mol. Biol..

[33] Bradford MM (1976). Rapid and sensitive method for the quantitation of microgram quantities of protein utilising the principle of protein dye binding. Anal. Biochem..

[34] Takashima, F. & Hibiya, T. An atlas of fish histology: normal and pathological features, second ed. Kodansha Ltd., Tokyo. pp 243 (1995).

[35] Smith SA (2019). Fish Diseases and Medicine.

[36] Nwani CD, Nnaji MC, Oluah SN, Echi PC, Nwamba HO, Ikwuagwu OE, Ajima MNO (2014). Mutagenic and physiological responses in the juveniles of African catfish, *Clarias gariepinus* (Burchell 1822) following short term exposure to praziquantel. Tissue Cell.

[37] Tavares-Dias M (2021). Toxicity, physiological, histopathological and antiparasitic effects of the formalin, a chemotherapeutic of fish aquaculture. Aquacult. Res..

[38] Blaxter JHS, Hoar WS, Randall RJ (1988). Pattern and variety in development. Fish Physiology.

[39] Stancova V, Plhalova L, Bartoskova M, Zivna D, Prokes M, Marsalek P, Blahova J, Skoric M, Svobodova Z (2014). Effects of mixture of pharmaceuticals on early life stages of tench (*Tinca tinca*). BioMed. Res. Int..

[40] Rico A, Phu TM, Satapornvanit K, Min J, Shahabuddin AM, Henriksson PJG, Murray FJ, Little DC, Dalsgaard A, Van den Brink PJ (2013). Use of veterinary medicines, feed additives and probiotics in four major internationally traded aquaculture species farmed in Asia. Aquacult..

[41] Tavares-Dias M (2021). Toxic, physiological, histomorphological, growth performance and antiparasitic effects of copper sulphate in fish aquaculture. Aquacult..

[42] McKim, J.M. Early life stage toxicity tests. in *Fundamentals of Aquatic Toxicology, Effects, Environmental Fate and Risk Assessment* (G.M. Rand, Ed.). (Taylor & Francis, 1995).

[43] Woltering DM (1984). The growth response in fish chronic and early life stage toxicity tests: a critical review. Aquat. Toxicol..

[44] Velisek J, Stara A, Kubec J, Zuskova E, Buric M, Kouba A (2020). Effects of metazachlor and its major metabolite metazachlor OA on early life stages of marbled crayfish. Sci. Rep..

[45] Velisek J, Stara A (2018). Effect of thiacloprid on early life stages of common carp (*Cyprinus carpio* L.). Chemosphere.

[46] Stepanova S, Dolezelova P, Plhalova L, Prokes M, Marsalek P, Skorica M, Svobodova Z (2012). The effects of metribuzin on early life stages of common carp (*Cyprinus carpio*). Pest. Biochem. Physiol..

[47] Sehonova P, Plhalovaa L, Blahova J, Berankova P, Doubkova V, Prokes M, Tichy F, Vecerek V, Svobodova Z (2016). The effect of tramadol hydrochloride on early life stages of fish. Environ. Toxicol. Pharmacol..

[48] Kubec J, Kouba A, Buřič M (2019). Communication, behaviour, and decision making in crayfish: A review. Zool. Anz..

[49] Ispir U, Kirici M, Yonar ME, Yonar MS (2017). Response of antioxidant system to formalin in the whole body of rainbow trout, Oncorhynchus mykiss. Cell. Mol. Biol.

[50] Sakin F, Yonar SM, Yonar ME, Saglam N (2012). Changes in selected immunological parameters and oxidative stress responses in different organs of *Oncorhynchus mykiss* exposed to ivermectin. Rev. Chem..

[51] Thomaz JM, Martins ND, Monteiro DA, Rantin FT, Kalinin AL (2009). Cardiorespiratory function and oxidative stress biomarkers in Nile tilapia exposed to the organophosphate insecticide trichlorfon (NEGUVON). Ecotoxicol. Environ. Safe..

[52] Yonar SM, Yonar ME, Pala A, Saglam N, Sakin F (2020). Effect of trichlorfon on some haematological and biochemical changes in *Cyprinus carpio*: The ameliorative effect of lycopene. Aquacult. Rep..

[53] Wolf JC, Wolfe MJ (2005). A brief overview of nonneoplastic hepatic toxicity in fish. Toxicol. Pathol..

[54] Farrell, G.C. & Larter, C.Z. Nonalcoholic fatty liver disease: from steatosis to cirrhosis. **43**, S99-112 (2006). 10.1002/hep.20973 16447287

[55] Purushotham A, Schug TT, Xu Q, Surapureddi S, Guo X, Li X (2009). Hepatocyte-specific deletion of SIRT1 alters fatty acid metabolism and results in hepatic steatosis and inflammation. Cell Metab..

